# Robust Visual-Inertial Odometry with Learning-Based Line Features in a Illumination-Changing Environment

**DOI:** 10.3390/s25165029

**Published:** 2025-08-13

**Authors:** Xinkai Li, Cong Liu, Xu Yan

**Affiliations:** James Watt School of Engineering, University of Glasgow, Glasgow G12 8QQ, UK; 3055752l@student.gla.ac.uk (C.L.); 2703613y@student.gla.ac.uk (X.Y.)

**Keywords:** visual-inertial odometry (VIO), simultaneous localization and mapping (SLAM), line features, deep learning

## Abstract

Visual-Inertial Odometry (VIO) systems often suffer from degraded performance in environments with low texture. Although some previous works have combined line features with point features to mitigate this problem, the line features still degrade under more challenging conditions, such as varying illumination. To tackle this, we propose DeepLine-VIO, a robust VIO framework that integrates learned line features extracted via an attraction-field-based deep network. These features are geometrically consistent and illumination-invariant, offering improved visual robustness in challenging conditions. Our system tightly couples these learned line features with point observations and inertial data within a sliding-window optimization framework. We further introduce a geometry-aware filtering and parameterization strategy to ensure the reliability of extracted line segments. Extensive experiments on the EuRoC dataset under synthetic illumination perturbations show that DeepLine-VIO consistently outperforms existing point- and line-based methods. On the most challenging sequences under illumination-changing conditions, our approach reduces Absolute Trajectory Error (ATE) by up to 15.87% and improves Relative Pose Error (RPE) in translation by up to 58.45% compared to PL-VINS. These results highlight the robustness and accuracy of DeepLine-VIO in visually degraded environments.

## 1. Introduction

Simultaneous Localization and Mapping (SLAM) serves as a foundational technology in robotics, enabling autonomous agents to perceive and navigate unknown environments without external positioning systems [1]. It plays a critical role in a wide range of applications, including autonomous driving [2], aerial drones [3], service robots [4], augmented reality (AR) [5], and planetary exploration [6]. Whether mapping an unfamiliar indoor space or navigating dynamic urban streets, SLAM provides the spatial awareness necessary for real-time decision-making and robust autonomy. SLAM is a core capability for autonomous systems operating in GPS-denied environments, such as indoors or underground [7,8]. Among various SLAM modalities, visual-inertial odometry (VIO) has emerged as a compelling solution due to its balance of accuracy, robustness, and hardware efficiency [1,9,10]. By tightly fusing image measurements with inertial data, VIO systems achieve accurate and drift-resilient pose estimation [11,12]. Representative works such as VINS-Mono [12], OKVIS [13], and MSCKF [11] have demonstrated strong performance in structured environments, particularly when abundant visual features are available.

However, these point-based methods often degrade in environments with weak textures, repetitive patterns, or dynamic illumination conditions frequently encountered in real-world scenarios such as corridors, tunnels, or dim indoor settings. Under such conditions, reliable keypoints are difficult to extract, and feature tracking becomes unstable [14]. To mitigate this issue, recent approaches have explored incorporating structural features such as lines into the SLAM pipeline. Line segments, which commonly exist in man-made environments, provide complementary geometric constraints and tend to remain stable under lighting variations [15,16,17]. PL-VINS [15] integrates line segments into VINS-Mono using LSD and LBD for detection and matching, respectively. It employs Plücker coordinates to represent spatial lines and jointly optimizes them with points and inertial measurements in a sliding-window framework. PLF-VINS [16] further introduces point-line coupled residuals and parallel line constraints to enhance robustness in structured scenes. Although these methods show promising improvements, they rely on handcrafted detectors that are sensitive to image noise, edge blur, and illumination changes, limiting their performance in challenging environments. Meanwhile, deep learning has advanced SLAM through learned features, depth, and motion priors [18]. DeepSLAM [19] and SP-Flow [20] demonstrate the feasibility of replacing traditional visual modules with neural networks to improve performance in degraded conditions. Inspired by this, we aim to enhance the robustness of VIO systems by learning illumination-invariant line representations.

In this paper, we present DeepLine-VIO, a robust visual-inertial odometry framework that leverages attraction-field-based learning for stable line extraction. Unlike prior work that directly uses traditional LSD for line detection [21], we employ a data-driven line representation that encodes both direction and proximity, enabling reliable feature detection under challenging visual conditions. Our system combines these learned line features with point features and inertial constraints in a tightly-coupled optimization framework. We further design a geometry-aware filtering and parameterization strategy to ensure consistent and accurate 3D reconstruction. Our main contributions are as follows:

(1) We present a novel learning-based visual-inertial odometry system that incorporates illumination-invariant line features trained via attraction fields.
(2) We develop an efficient filtering and matching pipeline that ensures geometric consistency of extracted lines, even under changing light and noisy conditions.
(3) We validate our method on challenging benchmark datasets with synthetic lighting perturbations, demonstrating significant improvements in trajectory accuracy and robustness over existing point- and line-based VIO systems.

## 2. Related Work

### 2.1. Visual-Inertial Odometry

Visual-inertial odometry (VIO) has been extensively studied in robotics and autonomous systems due to its ability to provide drift-resilient motion estimation in GPS-denied environments [22,23]. Traditional VIO methods can be broadly categorized into filter-based and optimization-based approaches. The Multi-State Constraint Kalman Filter (MSCKF) [11] is a representative filter-based method that achieves high computational efficiency by marginalizing out visual landmarks. In contrast, VINS-Mono [12] and OKVIS [13] adopt sliding-window nonlinear optimization to achieve higher accuracy through tighter visual-inertial coupling. These systems rely heavily on point features, which are effective in rich-textured environments but often fail in scenes with low contrast or repetitive patterns. Compared to VINS-Mono, which uses a monocular camera, ROVINS adopts a multi-fisheye omnidirectional camera setup and an IMU-assisted tracking strategy. It improves robustness under wide viewpoint changes and dynamic scenes. Although both use a sliding window graph optimization framework, ROVINS focuses more on system stability in its front-end design and data handling, aiming for reliable performance rather than maximum accuracy [24].

### 2.2. Line Features in SLAM and VIO

To address the limitations of point-based methods, several works have explored incorporating line features into the SLAM pipeline. Lines are abundant in structured environments, more robust under illumination changes, and provide complementary constraints to points. He et al. [25] proposed PL-VIO, a tightly-coupled visual–inertial odometry framework built from scratch. It jointly optimizes point and line features using reprojection residuals and IMU pre-integration in a sliding window, with Plücker coordinates representing spatial lines. Zhang et al. [26] proposed a line-enhanced visual-inertial SLAM method that applies bilateral filtering and geometric constraints for more efficient line processing. Their system optimized LSD parameters and used adaptive length thresholds, achieving faster matching and improved accuracy over PL-VIO and VINS-Mono, especially under weak textures and motion blur. Zhang et al. [27] proposed a visual SLAM optimization method that integrates both point and line features. The main innovation is the introduction of a ray-to-ray residual model for line feature optimization based on Plücker coordinates, which was incorporated into the ORB-SLAM3 framework.

PL-VINS [15] extends the open-source VINS-Mono by integrating line features into its pipeline. It introduces a practical engineering design for line extraction (based on LSD [21]), filtering, binary matching (LBD), and optimization within the existing graph-based framework. This makes it a reproducible and extendable system for incorporating structural features. Building on PL-VINS, PLF-VINS [16] introduces new residual models such as the Point-Line Coupled (PLC) residual, which leverages nearby lines to improve depth estimation of poorly triangulated points. It also adds parallel line constraints for structured scenes. Although these systems improve robustness in texture-sparse areas, their reliance on handcrafted detectors limits performance under visual degradation, such as low light or motion blur.

### 2.3. Deep Learning for SLAM and Feature Detection

Deep learning has shown promising results in various SLAM components, including depth estimation, pose regression, and feature extraction. Dynamic-SLAM integrates a convolutional neural network (CNN)-based SSD (Single Shot MultiBox Detector) into the ORB-SLAM2 framework to detect dynamic objects in real time, such as pedestrians and vehicles. The detection results are used to generate dynamic masks, which filter out feature points in these regions. This can prevent them from being used in front-end tracking and pose estimation, and finally helps reduce mismatches and localization failures caused by dynamic interference [28]. Similarly, Su et al. also introduced a parallel semantic thread, using the YOLOv5s deep learning detector to quickly identify dynamic objects in each frame, and combined optical flow analysis with optimized homography estimation to effectively remove dynamic feature points, thereby improving the perception of static environments [29].

DeepSLAM [19] replaces traditional geometric modules with learned depth and pose networks trained in an unsupervised manner. SP-Flow [20] proposes a self-supervised framework for learning keypoint locations and descriptors tailored for SLAM.

In the context of line features, recent works have explored deep representations that go beyond classical gradient-based detection. DeepLSD [30] introduces an attraction field representation that models line structures as continuous distance and orientation fields, improving robustness in low-texture and illumination-varying conditions. However, such learned line features have not been fully explored in tightly-coupled VIO systems.

### 2.4. Our Contribution

Our work bridges the gap between learning-based line detection and tightly-coupled VIO. Unlike prior systems that rely on fragile handcrafted line features [15,16], we incorporate a learned, attraction-field-driven line representation into a visual-inertial optimization framework. Furthermore, we introduce a novel filtering, parameterization, and matching strategy to ensure consistent integration of these line features in both the frontend and backend. Experimental results show that this combination significantly improves accuracy and robustness in visually degraded environments, particularly under dynamic lighting changes.

## 3. Methodology

### 3.1. System Overview

**Figure 1:**
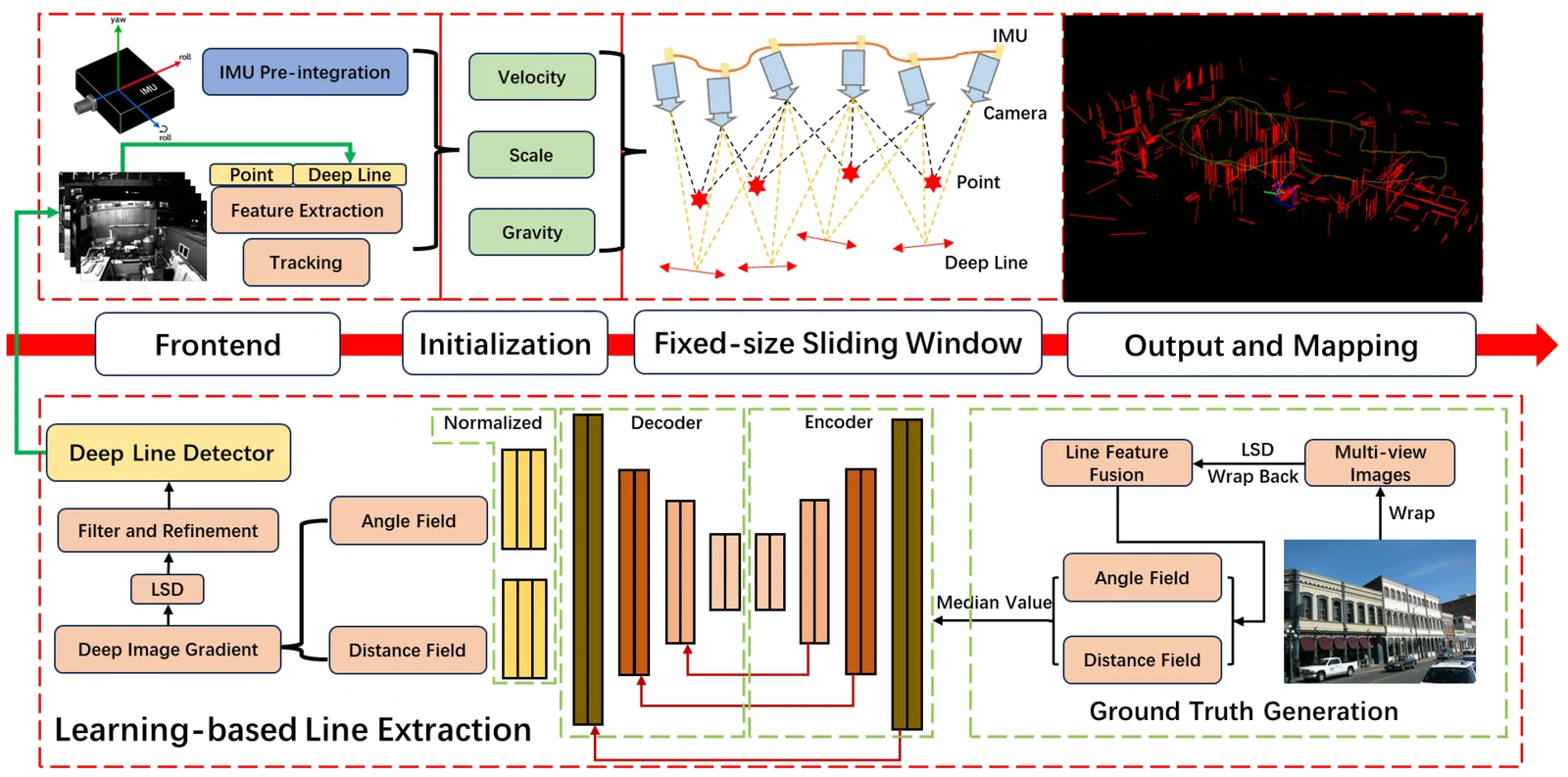
System Overview. The upper part illustrates the overall system pipeline, which is detailed in Section 2.1. The lower part presents the line feature detection process, which is elaborated in Section 2.2. In the mapping stage, the two trajectories correspond to the cases with and without loop closure. It should be noted that the loop closure part is used in this paper only for visualization purposes, and all evaluations, as a VIO system, are conducted on the trajectory without loop closure.

The system adopts a tightly-coupled visual-inertial estimation framework, Deepline-VIO, based on sliding window nonlinear optimization, which fuses point features, line features, and inertial measurements to achieve real-time, accurate, and robust state estimation. The overall pipeline consists of feature extraction and tracking, IMU pre-integration, visual-inertial initialization, sliding window optimization, and mapping. The overall system is shown in Figure 1, and each component is explained in detail below.

**A.**  **Feature Extraction and Tracking**

The front-end extracts sparse point and line features from the image sequence to provide stable and clean observations for subsequent optimization. Point features are tracked using the optical flow method to ensure trajectory continuity and accuracy. Line features are extracted using a Learning-based method, DeepLSD, which improves stability under low texture and poor lighting conditions. Details of the tracking process are provided in Section 2.3.

**B.**  **IMU Pre-integration Modeling**

To incorporate inertial measurements into the backend optimization efficiently, the system uses IMU pre-integration, which compresses continuous IMU data between two keyframes into compact and differentiable constraints. This module includes IMU noise and bias modeling, the definition of pre-integrated terms, and bias correction. The raw IMU measurements are modeled as follows. The accelerometer and gyroscope readings in the body frame are given by

(1) $${\hat{a}}_{t}=a_{t}+b_{a_{t}}+R_{w}^{t}g+n_{a_{t}},\;{\hat{\omega }}_{t}=\omega_{t}+b_{\omega_{t}}+n_{\omega_{t}}$$

where $b_{a_{t}}$ and $b_{\omega_{t}}$ are the accelerometer and gyroscope biases, $n_{a_{t}}$ and $n_{\omega_{t}}$ are zero-mean Gaussian noises, and g is the gravity vector in the world frame. In practice, both the accelerometer bias $b_{a_{t}}$ and gyroscope bias $b_{\omega_{t}}$ slowly drift over time due to temperature changes, sensor aging, and other factors. This drift is modeled as a random walk process driven by Gaussian noise, which enables the system to continuously correct for long-term bias variation during optimization. Based on this model, three types of pre-integrated terms are defined to describe the accumulated state change between time $t_{k}$ and $t_{k+1}$, and the position, velocity, and rotation pre-integration terms are defined as follows:

(2) $$\begin{array}{cc} \alpha_{b_{k}}^{b_{k+1}} & =\int_{t_{k}}^{t_{k+1}}\int_{t_{k}}^{s}R_{b}^{w}\left(s\right)\left(\hat{a}\left(s\right)-b_{a}\right)ds\;ds\\ \beta_{b_{k}}^{b_{k+1}} & =\int_{t_{k}}^{t_{k+1}}R_{b}^{w}\left(s\right)\left(\hat{a}\left(s\right)-b_{a}\right)ds\\ \gamma_{b_{k}}^{b_{k+1}} & =\int_{t_{k}}^{t_{k+1}}\frac{1}{2}\Omega \left(\hat{\omega }\left(s\right)-b_{\omega }\right)\gamma \left(s\right)\;ds \end{array}$$

where Ω(·) is the skew-symmetric matrix formed from angular velocity to represent small rotations. In the subsequent global nonlinear optimization of the sliding window, the bias terms $b_{a}$ and $b_{\omega }$ are updated iteratively. To avoid re-integrating IMU measurements every time, the system uses a first-order correction method to adjust the pre-integrated results with Jacobians:

(3) $$\begin{array}{cc} {\hat{\alpha }}_{b_{k}}^{b_{k+1}} & =\tilde{\alpha }+J_{\alpha }^{b_{a}}\delta b_{a}+J_{\alpha }^{b_{\omega }}\delta b_{\omega }\\ {\hat{\beta }}_{b_{k}}^{b_{k+1}} & =\tilde{\beta }+J_{\beta }^{b_{a}}\delta b_{a}+J_{\beta }^{b_{\omega }}\delta b_{\omega }\\ {\hat{\gamma }}_{b_{k}}^{b_{k+1}} & =\tilde{\gamma }+J_{\gamma }^{b_{\omega }}\delta b_{\omega } \end{array}$$

This strategy allows for efficient bias correction in each iteration without repeating the costly integration process. As a result, the IMU constraints can be updated quickly and accurately, improving the overall performance of the optimization.

**C.**  **Visual-Inertial Initialization**

Before the sliding-window optimization begins, the system performs a visual-inertial initialization to estimate the initial velocity, gravity vector, and real-world scale. This module operates in a vision-only manner and uses structure-from-motion (SfM) inside the sliding window to reconstruct a sparse camera trajectory and 3D landmarks under an arbitrary scale. These results are then temporally aligned with IMU preintegration to build a unified reference frame. The initialization provides accurate priors for the subsequent tightly-coupled optimization. Next, the system estimates the gyroscope bias by comparing the IMU-integrated rotation $\gamma_{b_{k}}^{b_{k+1}}$ with the rotation from SfM ${\hat{\gamma }}_{b_{k}}^{b_{k+1}}$. The error is minimized by solving:

(4) $$\min_{\delta b_{\omega }}\sum_{k\in B}{∥{\left(\gamma_{b_{k}}^{b_{k+1}}\right)}^{-1}{\hat{\gamma }}_{b_{k}}^{b_{k+1}}∥}^{2}$$

After bias correction, all IMU pre-integration terms are updated. With rotation known, a linear least-squares problem is solved to estimate body-frame velocity v, gravity vector $g^{c_{0}}$, and scale *s*:

(5) $$\min_{X_{I}}\sum_{k\in B}{∥{\hat{z}}_{b_{k}}^{b_{k+1}}-H_{b_{k}}^{b_{k+1}}X_{I}∥}^{2}$$

where $X_{I}=\left\{v_{b_{0}}^{b_{0}},\cdots ,v_{b_{n}}^{b_{n}},g^{c_{0}},s\right\}$ includes the velocity of each frame, gravity vector, and scale. Next, the gravity vector has only two degrees of freedom (its norm is fixed), so we refine it by applying a perturbation in its tangent space. Let the current gravity be $\hat{g}$, we express it as:

(6) $$g=\hat{g}+w_{1}b_{1}+w_{2}b_{2}$$

where $b_{1},b_{2}$ are two orthogonal basis vectors spanning the tangent plane. Finally, the gravity direction is rotated to align with the Z-axis of the world frame. All poses and velocities are transformed into the world frame, and the visual trajectory is scaled by the estimated factor. At this point, the system has a consistent reference frame with known scale, gravity, and initial motion, ready for sliding window optimization.

**D.**  **Sliding Window Optimization and Residual Modeling**

To achieve real-time and high-accuracy estimation, the system maintains a fixed-size sliding window of recent states, including several keyframes and their associated IMU and visual measurements(points and lines). The oldest states are marginalized out when new keyframes are inserted, while their information is retained as a prior. At each time step, a nonlinear optimization is performed over the active window, incorporating multiple types of constraints. Specifically, the objective function minimizes a weighted sum of residuals, including IMU preintegration errors, visual reprojection errors, and historical priors. The formulation is:

(7) $$\min_{X}\left\{{∥r_{\mathrm{prior}}∥}^{2}+\sum {∥r_{\mathrm{imu}}∥}^{2}+\sum \rho \left({∥r_{\mathrm{point}}∥}^{2}\right)+\sum \rho \left({∥r_{\mathrm{line}}∥}^{2}\right)\right\}$$

Here, X denotes the set of all state variables, including pose, velocity, bias, inverse depth of feature points, and spatial line parameters. ρ(·) is the Huber kernel function, which improves robustness to outliers. The meaning and role of each residual are explained as follows.

Prior residual $r_{\mathrm{prior}}$: As the sliding window shifts, older frames are marginalized to control variable size. To preserve the useful information of removed frames, the system constructs a prior residual. A smaller prior residual means the current estimate agrees with the history. This term ensures estimation continuity and prevents sudden jumps.

IMU residual $r_{\mathrm{imu}}$: The IMU works at a much higher frequency than the camera. To avoid repeated integration during optimization, IMU measurements are pre-integrated between two keyframes into three components: α: relative position change β: relative velocity change γ: relative rotation change. These pre-integrated quantities are compared with the motion predicted by the current state estimate. A small residual indicates that the optimized motion is consistent with the IMU measurement, meaning the result is physically reasonable. This residual also helps recover the world scale and supports the system when visual features are missing.

Point feature residual $r_{\mathrm{point}}$: Each feature point is parameterized by inverse depth in the first observed frame. In other frames, its re-projection is computed using the estimated pose and compared to the image observation. This residual measures the difference between predicted and observed pixel positions. Since the re-projection depends on the relative pose between frames, the point residual imposes constraints on both translation and rotation of the camera. A smaller residual means the current pose estimation explains the image observations well.

**Line feature residual $r_{\mathrm{line}}$**: In low-texture or repeated-pattern scenes, point tracking often becomes unreliable. To address this, the system introduces spatial lines as complementary constraints. The construction of spatial lines follows these steps: Starting from the matched line segments in the two frames (such as the edge of a straight line), combined with the camera poses of the two frames, using triangulation to obtain a 3D line $L_{w}$, represented by Plücker coordinates $\left(n_{w},d_{w}\right)$, where $d_{w}$ is the direction of the line and $n_{w}$ encodes its moment with respect to the origin. Plücker coordinates have 6 elements but only 4 degrees of freedom for a spatial line, so they are redundant. To solve this, the line is reparameterized into a minimal orthonormal form σ=(θ,τ), where θ is the rotation vector and τ adjusts the line’s position in the normal plane. This compact representation is better for optimization. During optimization, the spatial line $L_{w}$ is first transformed from the world frame to the camera frame $c_{i}$, and then projected onto the image plane to obtain a predicted image line. The line residual is constructed by measuring the perpendicular distance from the midpoint of the observed image line segment $z_{L_{j}}^{c_{i}}$ to this predicted line. In the formula, rL(·) calculates this signed distance error, which is then weighted by the observation covariance $\Sigma_{L_{j}}^{c_{i}}$ and passed through the robust Huber kernel ρ(·) to suppress outliers.

(8) $$r_{\mathrm{line}}=\sum_{(i,j)\in L}\rho \left({∥r_{L}\left(z_{L_{j}}^{c_{i}},X\right)∥}_{\Sigma_{L_{j}}^{c_{i}}}^{2}\right)$$

This residual measures the perpendicular distance between the predicted spatial line and the midpoint of the observed line segment in the image. A smaller residual means the spatial line matches well with the observed geometry in the image. To ensure stable optimization and maintain the orthogonality of the line representation, the system adopts an orthonormal parameterization and updates it using Lie algebra on the tangent space. This strategy preserves the geometric structure of the parameters and improves convergence robustness. By jointly minimizing all residuals, the system fuses information from history, IMU, visual points, and spatial lines. The unified framework ensures accurate, continuous, and physically consistent state estimation, even under challenging environments.

### 3.2. Learning-Based Line Extraction

This method combines the advantages of deep prediction and handcrafted detectors, and can stably output structured line segments under complex illumination and weak texture conditions, while taking into account positioning accuracy and computational efficiency. It introduces the representation of Attraction Field, modeling discrete line segments as continuous fields and using neural networks to predict the distance and direction from each pixel to its nearest line segment. Specifically, it has two continuous components: the distance field D(u,v) and the direction field A(u,v). Among them, *D* represents the Euclidean distance from the pixel to the nearest line segment, and *A* represents the direction of the corresponding line segment, defined as:

(9) $$D=\sqrt{x^{2}+y^{2}},\;A=\arctan\frac{y}{x}+\frac{\pi }{2}\;\;\;\mathrm{mod}\;\pi .$$

To generate pseudo labels for the attraction field, the input image is warped with multiple random homographies to simulate different views. Line segments are extracted by LSD from these warped images and re-projected back to the original image. A median fusion is then performed at the pixel level to produce robust attraction field supervision. This method does not rely on manual annotations and is suitable for training on arbitrary image data. After obtaining the pseudo-labels, the network takes the image as input and predicts two attraction field components for each pixel: the distance field $\hat{D}\left(u,v\right)$ and the angle field $\hat{A}\left(u,v\right)$. The distance field represents the pixel-wise Euclidean distance to the nearest line segment, while the angle field encodes the direction of that line segment in the range [0,π]. The network adopts a U-Net encoder-decoder architecture to capture multi-scale features, and produces two output channels of the same resolution as the input image, corresponding to the normalized distance and angle fields. To stabilize distance regression, the raw distance value *D* is first logarithmically normalized:

(10) $$D_{n}=-\log\left(\frac{D}{r}\right)$$

where *r* is the effective radius around each line, typically set to 5 pixels. The network is trained to predict the normalized distance ${\hat{D}}_{n}$, and the original distance can be recovered by inverse exponential transformation:

(11) $$\hat{D}=r\cdot e^{-{\hat{D}}_{n}}$$

To represent line orientation, the network also predicts an angle field. The angle $\hat{A}\left(u,v\right)$ is computed by applying a sigmoid activation followed by a linear mapping:

(12) $$\hat{A}\left(u,v\right)=\pi \cdot \mathrm{sigmoid}\left(x\right)$$

this guarantees the predicted angle lies within [0,π], and avoids the ambiguity of direction flipping due to the periodic nature of line orientation. The final loss function combines the L1 loss on the normalized distance and a periodic-aware L2 loss on the angle:

(13) $$L=∥{\hat{D}}_{n}-D_{n}{∥}_{1}+\min\left(∥\hat{A}{-A∥}_{2},\;∥\pi -|\hat{A}-A{|∥}_{2}\right)$$

To extract structured line segments, the predicted distance and angle fields are converted into a gradient-like representation, referred to as a deep image gradient, and fed into the LSD detector to acquire line segments. However, there are some unstable or geometrically inconsistent lines, so an attraction field-based filtering step is applied after initial LSD detection. For each detected line, a fixed number of points are uniformly sampled. At each point $p_{i}$, the predicted distance $\hat{D}\left(p_{i}\right)$ and angle $\hat{A}\left(p_{i}\right)$ from the network are queried. A point is regarded as consistent if both the distance error is below a threshold $\eta_{D}$ and the angle deviation is below $\eta_{\theta }$. If fewer than 50% of the points on a line meet these conditions, the entire line is discarded. This filtering strategy effectively removes false positives caused by weak responses, double-edge ambiguity, or structural noise. After filtering, to improve the geometric accuracy of detected line segments, a sub-pixel refinement is applied to jointly optimize the center position $m_{l}$ and direction $\theta_{l}$ of each segment. This refinement adjusts the line segments to better conform to the predicted attraction field and the structural layout of vanishing points. The optimization objective consists of three terms:

(14) $$C=\lambda_{A}C_{A}+\lambda_{D}C_{D}+\lambda_{V}C_{V}$$

where $C_{A}$, $C_{D}$, and $C_{V}$, respectively, represent the angular consistency with the direction field, the distance field response of sampled points, and the deviation from the direction of vanishing points. A smaller value of C indicates better geometric alignment between the line segment and the predicted scene structure. The optimized results form the final enhanced line segments, completing the overall line detection process.

### 3.3. DeepLine-Enhanced Visual-IMU Odometry with Efficient Line Filtering and Matching

**Figure 2:**
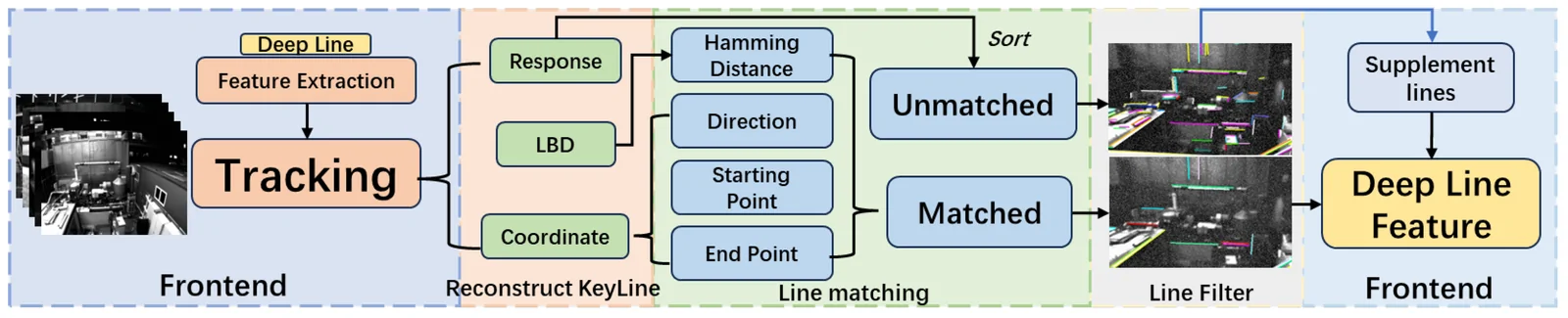
Line feature tracking and matching. Integration process of line features with VIO. In the line filtering stage, after screening the detected line features in the scene, the cluttered lines in the image are reduced.

The original PL-VINS uses the gradient-based LSD algorithm to extract line segments as the line feature. LSD is a line segment detection algorithm that does not require parameter adjustment and has a fast calculation speed. It has strong false detection control ability. It can accurately extract the real straight line structure in the image and is widely used in scenes such as visual navigation, image registration, remote sensing analysis, etc. [21]. However, under extreme conditions such as lighting changes, LSD cannot detect enough line segments, resulting in the loss of line information. To enhance the quality of line segment detection, we replace the traditional LSD module with DeepLSD, a learning-based detector that leverages attraction fields to encode geometric proximity and orientation. Compared to conventional methods, DeepLSD generates structurally coherent gradient representations that serve as robust input to LSD. This significantly improves edge localization and reduces sensitivity to noise, texture sparsity, and local gradient inconsistencies, resulting in more stable and accurate line detection [30], Our process for this part is shown in Figure 2 and will be explained in detail below.

DeepLSD is used as a front-end detector to replace LSD and extract line segment information from images. However, since it returns the line segment coordinate value, in order to directly apply its output, the original line segment matching logic needs to be adjusted. First, the KeyLine structure needs to be reconstructed. KeyLine was originally a structure returned directly by LSD to VINS. It contains rich information about the detected line segment for subsequent line segment matching. Among them, LSD provides gradient-based response values, while Deeplsd does not output or directly use explicit gradient calculations. It embeds the gradient information in the deep learning network as part of the feature extraction in the network to optimize the final output. Therefore, we need to introduce a normalized response indicator here:

(15) $$r=\frac{\mathrm{lineLength}}{\max(W,H)}$$

This value reflects the relative scale of the line segment in the image, where *W* and *H* are the width and height of the image. Longer line segments are generally more geometrically stable and can provide stronger constraints in the back-end optimization. In the original LSD detection process, in order to improve the robustness to multi-scale structures, an image pyramid is constructed, and the LSD algorithm is run on each layer of the image to extract line segments. The image pyramid is a set of multiresolution images formed by reducing the original image layer by layer. Its main function is to avoid extracting only short or local line segments at the original image scale, thus improving overall detection integrity. However, in DeepLSD, since the deep neural network itself has multi-scale perception capabilities, the network has already modeled the scale information of the image structure internally, so there is no need to explicitly build an image pyramid. Therefore, we also adjusted the system structure during the process of connecting to DeepLSD: no longer building an image pyramid, only retaining line segment information at a single scale. This simplification not only reduces computational overhead but also better fits the design logic of the DeepLSD model.

After constructing the KeyLine structure, each line segment is described using the Line Band Descriptor (LBD), which encodes local appearance into a binary string. Matching is initially performed by computing the Hamming distance between LBD descriptors; a smaller distance indicates higher similarity and a greater likelihood that the two segments correspond to the same physical line. To reduce mismatches caused by the increased number of line segments produced by DeepLSD, the Hamming distance threshold is lowered from the original value of 30 to 28, retaining only matches with minimal descriptor differences. In addition, geometric constraints are applied, requiring matched segments to have small endpoint displacement and angular deviation, thereby ensuring spatial and directional consistency. These three criteria, descriptor similarity, endpoint proximity, and directional alignment, together constitute the core logic of the line segment matching process.

To avoid sparse features in the direction, it is necessary to divide the unmatched new line segments into two categories: horizontal lines and vertical lines according to their direction angles, and count the number of each type of line segment currently tracked. If the number of lines in a certain direction is less than 35, the line filling operation will be triggered. In the line filling stage, the unmatched line segments in the corresponding direction must first be sorted according to the normalized response value, and several line segments with the highest response values are selected to supplement them to ensure the stability of the geometric structure. Since DeepLSD has already learned the gradient information in the deep learning network, it further screens based on the size of the normalized response index to achieve “selecting the best from the best”.

In the tracking stage, we retain the original residual modeling and optimization strategy from PL-VINS, including the use of Plücker coordinates and orthonormal parameterization for line features. The updated line segments extracted by DeepLSD are seamlessly integrated into the existing graph structure without modifying the cost formulation. This allows the system to benefit from higher-quality and more stable line observations, improving accuracy and robustness while preserving the overall optimization framework.

## 4. Experimental Results

In this chapter, we evaluate the performance of Deepline-VIO, including both accuracy and real-time capability. Because our system is VIO, all evaluation metrics in this paper are computed without loop closure to fairly reflect the front-end performance. However, for further discussion, loop closure mapping will be used for visualization at the end. All experiments are conducted on a system running Ubuntu 18.04 with ROS Melodic, equipped with an AMD Ryzen 9 7945HX CPU and an NVIDIA GeForce RTX 4060 Laptop GPU.

### 4.1. Accuracy

In order to demonstrate the advantages of DeepLine-VIO, we performed a number of targeted perturbations on the input image to simulate some extreme environments. First, we simulate low-light conditions by applying overall darkening and gamma correction to emphasize detail degradation in dark regions. Gaussian blur and slight random noise are then added to mimic the blurring and noise interference introduced by real sensors during imaging. In addition, a periodic brightness perturbation mechanism is introduced to dynamically vary the contrast and brightness across frames, reflecting inter-frame illumination fluctuations. Based on these perturbations, we compare the original method using LSD with the improved method incorporating DeepLSD. Experiments are conducted on the EuRoC MAV Dataset (European Robotics Challenge Micro Aerial Vehicle Dataset), and performance is evaluated using the root mean square error (RMSE) of the Absolute Trajectory Error (ATE) and Relative Pose Error (RPE). However, the EuRoC dataset is all in artificial environments and has a small range of motion. To compensate for the limitations of the EuRoC dataset, we also conducted experimental verification on the TUM-VI outdoor dataset. This dataset has a larger range and contains rich natural lighting variations, and does not require additional processing. In this dataset, we focused on trajectory absolute accuracy and used ATE RMSE for evaluation.

For ATE, it reflects the absolute accuracy of the final estimated trajectory. As shown in Table 1, the accuracy improves by 15.87%, 14.85%, 11.41%, and 11.82% on the four difficult sequences, respectively. This indicates that under more challenging conditions, the method using DeepLSD provides more stable line feature extraction, leading to higher trajectory accuracy. On the medium and easy sequences, the improvement is not significant, as visual information plays a limited role in correcting SLAM in simpler environments, and the degradation of line features has less impact on the final result. As shown in Table 2, in the large-scale, long-duration TUM-VI outdoor dataset, where illumination changes are frequent and random in natural environments, our method achieves significant improvements in most sequences, further confirming the above analysis.

**Figure 3:**
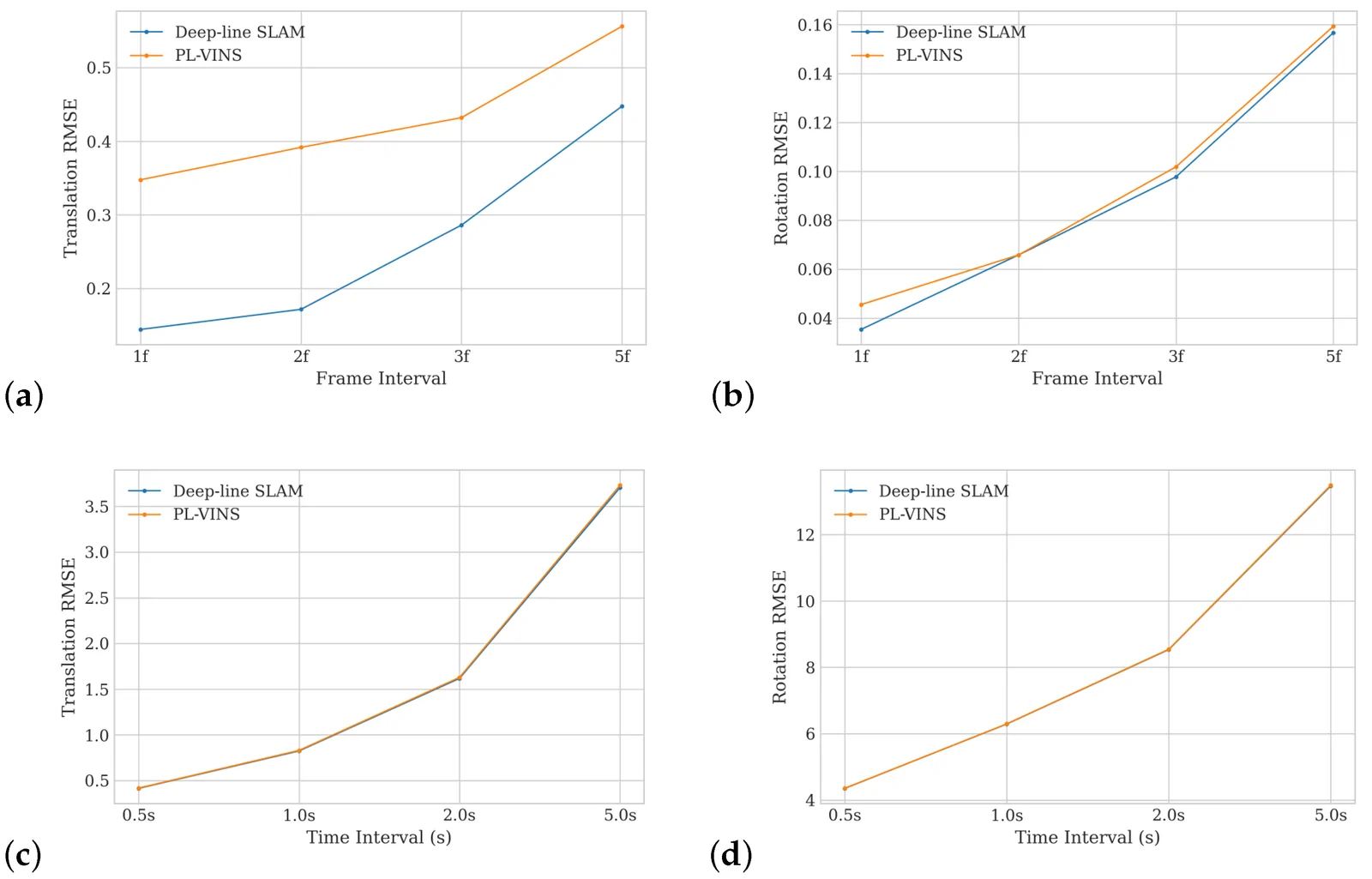
Relative errors under different intervals on MH_05_difficult. (**a**,**b**): 1-frame interval. (**c**,**d**): 5-s interval.

For RPE, it measures the deviation in rotation and translation between pose pairs. Compared with Absolute Trajectory Error (ATE), it better reflects the local smoothness and consistency of the UAV during motion. When calculating RPE, a fixed evaluation interval must be specified. In this paper, we adopt two types of evaluation methods: frame interval and time interval. The frame interval method (e.g., 1 f, 2 f, 5 f) evaluates the error between poses with a fixed number of frames in between, and focuses more on the relative stability at the step level of the trajectory; the time interval method (e.g., 0.5 s, 1.0 s, 5.0 s) evaluates error based on fixed time differences, which is closer to actual runtime behavior. In general, the frame interval method emphasizes motion error in short moments, while the time interval method better reflects long-term consistency. In Figure 3, RPE is evaluated on the dataset MH_05_difficult using different frame and time intervals. It can be observed that the longer the interval, the larger the accumulated error. Therefore, we select a 1-frame interval to evaluate the entire dataset and reflect instantaneous error, and a 5-s interval to reflect the relative error over the longest possible duration. For a fixed 1-frame rate, DeepLine-VIO shows a clear improvement in accuracy for the Trans RMSE on all difficult sequences in Table 3. However, for the Rot RMSE on the V2_03_difficult and MH_04_difficult sequences, it performs slightly worse, which differs from the improvement observed in ATE. A possible explanation is that the saturated line features in this sequence continuously corrected the pose, maintaining high accuracy in ATE, but the excessive corrections may have negatively affected the local motion smoothness within each one-frame interval. Similar to ATE, the difference between the two methods is negligible on the medium and easy sequences. In addition, at a fixed time of 5 s, the results of the two methods are similar in Table 4, which indicates that over a longer time period, frequent trajectory corrections with rich line feature constraints do not lead to a decrease in long-term stability.

**Figure 4:**
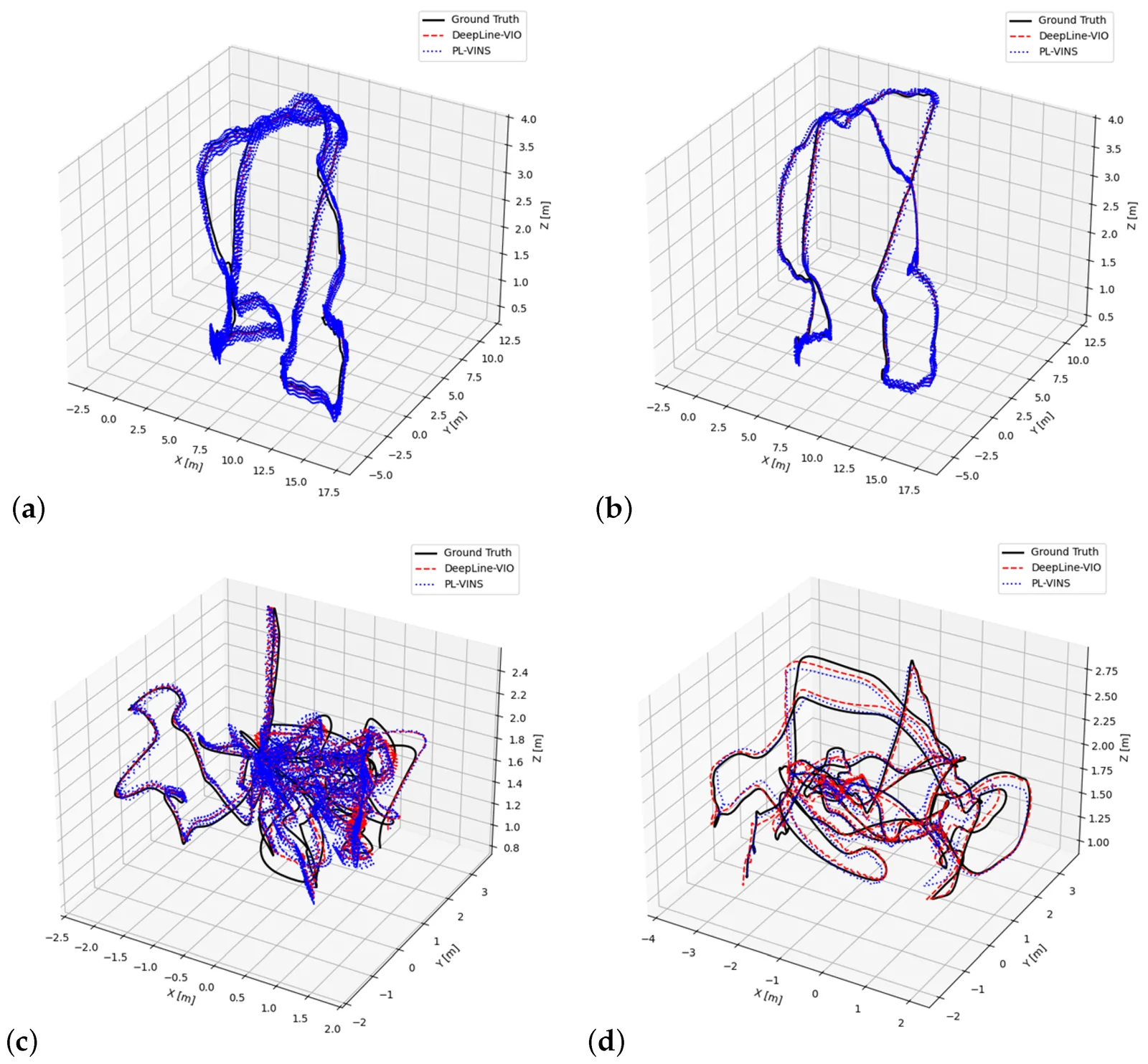
Comparison of estimated trajectories on the four difficult sequences. (**a**) MH_05_difficult. (**b**) MH_04_difficult. (**c**) V1_03_difficult. (**d**) V2_03_difficult.

**Figure 5:**
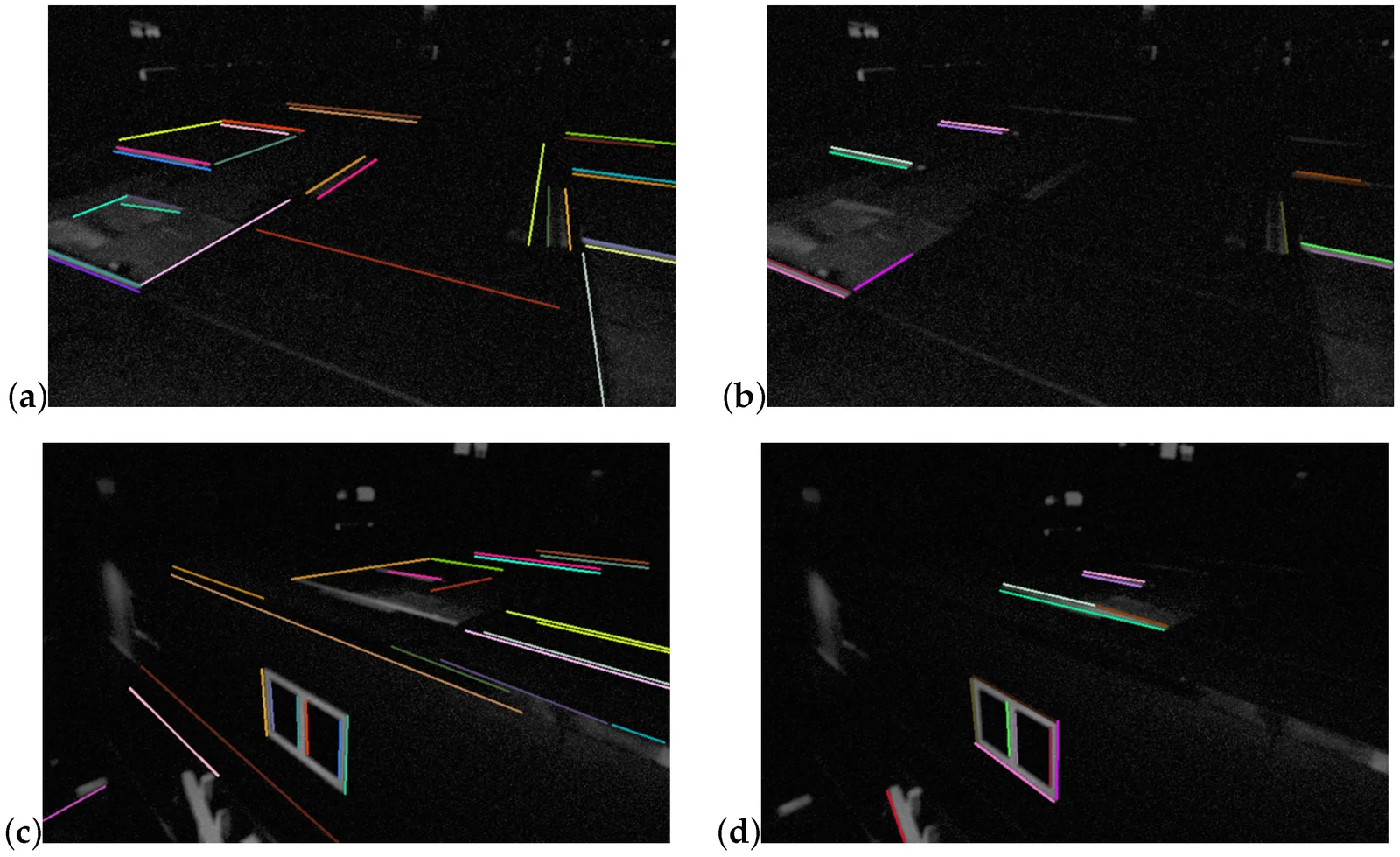
Comparison of Front-End Detection Results(Lines at the edge of the scene) under Low-Brightness Conditions. (**a**,**c**) DeepLine-VIO result. (**b**,**d**) PL-VINS result.

**Figure 6:**
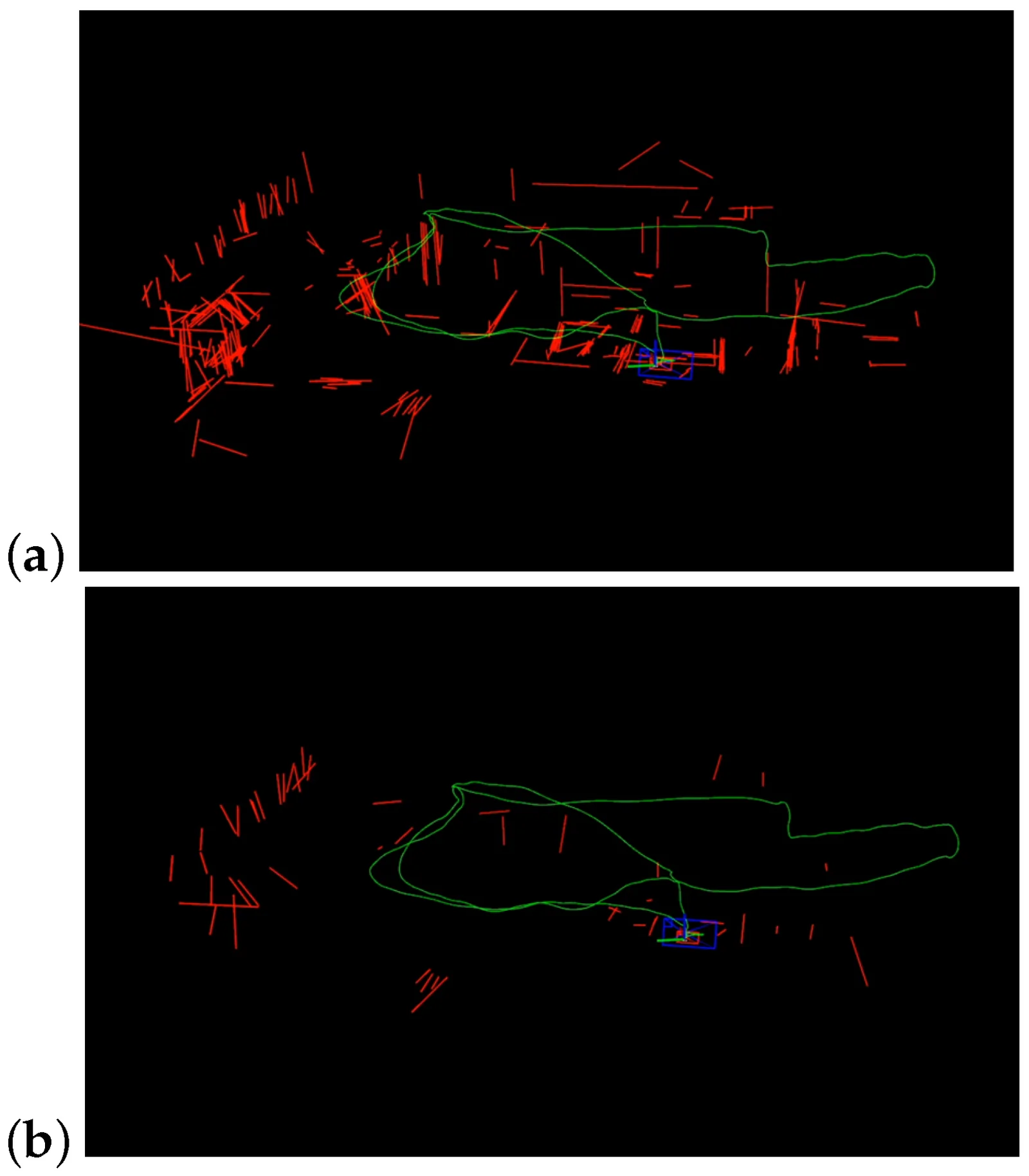
Comparison of mapping results under Illumination Variations. (**a**) DeepLine-VIO Mapping. (**b**) PL-VINS Mapping.

Figure 4 shows the estimated 3D trajectories of the two methods on difficult sequences. In the first three subfigures, it is clear that the blue trajectory (ours) follows the ground truth more closely, while the red trajectory diverges more, indicating that DeepLine-VIO achieves significantly higher accuracy under extreme conditions. As shown in Figure 5, under the same extremely low-light environment, PL-VINS struggles to detect edge information from the scene, while DeepLine-VIO can extract more line features from the images. This is also reflected in Table 5, where DeepLine-VIO detects more line features at the front end. This difference appears again in the loop-closure mapping results shown in Figure 6 (loop closure is only used for map visualization here and does not participate in trajectory evaluation). In this 3D mapping space, the spatial lines from the original PL-VINS gradually degrade: its front end cannot stably extract enough line features, and the lack of consistent structure between adjacent frames leads to failed line matching and unreliable triangulation. As a result, those missing lines cannot be added to the map during loop closure, making the final map sparse. In contrast, DeepLine-VIO produces a more complete map, showing that its front end retains enough reliable line features to resist frequent lighting changes and low-brightness interference.

### 4.2. Real-Time Analysis

As shown in Table 6, while the deep learning-based line detector significantly increases the time cost of line feature extraction compared to the original manual detector, the processing time per thread remains below 100 ms. Therefore, the system can maintain its original operating frequency without downsampling, indicating that the introduction of the deep learning model does not reduce its real-time performance.

## 5. Conclusions

In this paper, we presented DeepLine-VIO, a robust visual-inertial odometry system that enhances trajectory estimation under challenging illumination conditions by leveraging learned structural line features. Unlike prior methods that rely on handcrafted line detectors, our approach employs a data-driven attraction field representation to extract semantically consistent and geometrically reliable lines, improving visual robustness in texture-degraded or low-light environments.

We integrate these line features with point observations and inertial measurements in a tightly-coupled sliding-window optimization framework. A geometry-aware filtering and parameterization strategy ensures that only meaningful line segments contribute to state estimation. Experimental results on the EuRoC dataset, under synthetic lighting perturbations, demonstrate that our method consistently outperforms existing point- and line-based VIO systems in both accuracy and robustness.

Importantly, all evaluations are conducted without loop closure, highlighting the practical effectiveness of the proposed frontend enhancements in real-time, GPS-denied navigation scenarios. In future work, we plan to extend this framework to support semantically-aware line selection, real-time deployment on embedded platforms, and reduce the computational cost of deep learning-based line detectors through model compression techniques such as network pruning, quantization, or knowledge distillation. Furthermore, we plan to introduce scene-aware joint optimization or mutual improvement schemes based on the learning process of line features.

## Figures and Tables

**Table 1:** ATE RMSE comparison between PL-VINS and DeepLine-VIO on EuRoC sequences under illumination-changing conditions.

Dataset	PL-VINS	DeepLine-VIO	Improvement (%)
MH_05_difficult	0.3329	0.2801	15.87
MH_04_difficult	0.2707	0.2305	14.85
V1_03_difficult	0.1663	0.1473	11.41
V2_03_difficult	0.2412	0.2127	11.82
MH_03_medium	0.2320	0.2254	2.86
V1_02_medium	0.1249	0.1243	0.49
V2_02_medium	0.1375	0.1284	6.67
MH_02_easy	0.1621	0.1618	0.15
MH_01_easy	0.1473	0.1473	0.04
V1_01_easy	0.0744	0.0737	0.92

**Table 2:** ATE RMSE comparison between PL-VINS and DeepLine-VIO on TUM-VI Outdoors sequences.

Dataset	PL-VINS	DeepLine-VIO	Improvement (%)
Outdoors1	58.6819	55.3845	5.62
Outdoors2	118.3505	114.9687	2.86
Outdoors3	25.0901	21.6994	13.51
Outdoors4	9.5543	9.6601	−1.11
Outdoors5	24.8762	15.0594	39.46
Outdoors6	133.5429	118.7678	11.06
Outdoors7	33.0223	31.2352	5.41
Outdoors8	24.2436	24.0311	0.88

**Table 3:** RPE RMSE comparison and relative changes between PL-VINS and DeepLine-VIO (1-frame interval).

Dataset	PL-VINS		DeepLine-VIO		Change	
	Trans_RMSE	Rot_RMSE	Trans_RMSE	Rot_RMSE	Trans_RMSE	Rot_RMSE
MH_05_difficult	0.3477	0.0455	0.1445	0.0354	58.43%	22.30%
MH_04_difficult	0.2283	0.0497	0.1462	0.0550	35.95%	−10.56%
V1_03_difficult	0.1694	0.1306	0.1319	0.1031	22.14%	21.03%
V2_03_difficult	0.1090	0.1562	0.1088	0.1620	0.18%	−3.71%
MH_03_medium	0.1641	0.0469	0.1617	0.0618	1.44%	−31.79%
V1_02_medium	0.1453	0.1706	0.1453	0.1706	0.00%	0.00%
V2_02_medium	0.1209	0.1024	0.1195	0.1200	1.19%	−17.13%
MH_02_easy	0.0780	0.0627	0.0780	0.0627	0.00%	0.00%
MH_01_easy	0.0801	0.0635	0.0801	0.0635	−0.01%	0.00%
V1_01_easy	0.0751	0.0876	0.0751	0.0876	−0.01%	0.00%

**Table 4:** RPE RMSE comparison and relative changes between PL-VINS and DeepLine-VIO (5-s interval).

Dataset	PL-VINS		DeepLine-VIO		Change	
	Trans_RMSE	Rot_RMSE	Trans_RMSE	Rot_RMSE	Trans_RMSE	Rot_RMSE
MH_05_difficult	3.7319	13.4837	3.7060	13.4624	−0.69%	−0.16%
MH_04_difficult	4.0944	13.9614	4.0567	13.9507	−0.92%	−0.08%
V1_03_difficult	0.1380	27.7495	0.1428	27.7437	3.47%	−0.02%
V2_03_difficult	4.9929	28.4594	5.0185	28.3558	0.51%	−0.36%
MH_03_medium	8.1860	15.7445	8.1816	15.7446	−0.05%	0.00%
V1_02_medium	1.2568	26.4574	1.2578	26.4575	0.08%	0.00%
V2_02_medium	4.5907	26.3838	4.5908	26.4043	0.00%	0.08%
MH_02_easy	4.6397	14.8012	4.6396	14.8014	0.00%	0.00%
MH_01_easy	3.8185	14.0366	3.8184	14.0363	0.00%	0.00%
V1_01_easy	0.4343	20.1465	0.4341	20.1458	−0.05%	0.00%

**Table 5:** Average number of line features detected per frame and relative change.

Dataset	PL-VINS	DeepLine-VIO	Improvement (%)
MH_05_difficult	22.6305	32.9479	45.59
MH_04_difficult	23.7498	30.6881	29.21
V1_03_difficult	16.0668	18.3584	14.26
V2_03_difficult	15.0747	16.8990	12.10

**Table 6:** Comparison of Average Processing Time between PL-VINS and Deepline-VINS.

Threads	Modules	Times (ms)		System Frame Rate (Hz)	
		PL-VINS	Deepline-VIO	PL-VINS	Deepline-VIO
1	Point Detection and Tracking	6.1	6.01		
	Line Detection	5.3	44.5	10	10
	Line Tracking	9.3	9.61		
2	Local VIO	43.2	43.8	10	10

## Data Availability

The original contributions presented in this study are included in the article. Further inquiries can be directed to the corresponding author.
